# Infection with and Carriage of *Mycoplasma pneumoniae* in Children

**DOI:** 10.3389/fmicb.2016.00329

**Published:** 2016-03-23

**Authors:** Patrick M. Meyer Sauteur, Wendy W. J. Unger, David Nadal, Christoph Berger, Cornelis Vink, Annemarie M. C. van Rossum

**Affiliations:** ^1^Department of Pediatrics, Division of Pediatric Infectious Diseases and Immunology, Erasmus MC–Sophia Children’s Hospital, University Medical CenterRotterdam, Netherlands; ^2^Laboratory of Pediatrics, Division of Pediatric Infectious Diseases and Immunology, Erasmus MC–Sophia Children’s Hospital, University Medical CenterRotterdam, Netherlands; ^3^Division of Infectious Diseases and Hospital Epidemiology, and Children’s Research Center, University Children’s Hospital of ZurichZurich, Switzerland; ^4^Erasmus University College, Erasmus UniversityRotterdam, Netherlands

**Keywords:** *Mycoplasma pneumoniae*, pneumonia, carriage, children, diagnosis

## Abstract

“Atypical” pneumonia was described as a distinct and mild form of community-acquired pneumonia (CAP) already before *Mycoplasma pneumoniae* had been discovered and recognized as its cause. *M. pneumoniae* is detected in CAP patients most frequently among school-aged children from 5 to 15 years of age, with a decline after adolescence and tapering off in adulthood. Detection rates by polymerase chain reaction (PCR) or serology in children with CAP admitted to the hospital amount 4–39%. Although the infection is generally mild and self-limiting, patients of every age can develop severe or extrapulmonary disease. Recent studies indicate that high rates of healthy children carry *M. pneumoniae* in the upper respiratory tract and that current diagnostic PCR or serology cannot discriminate between *M. pneumoniae* infection and carriage. Further, symptoms and radiologic features are not specific for *M. pneumoniae* infection. Thus, patients may be unnecessarily treated with antimicrobials against *M. pneumoniae*. Macrolides are the first-line antibiotics for this entity in children younger than 8 years of age. Overall macrolides are extensively used worldwide, and this has led to the emergence of macrolide-resistant *M. pneumoniae*, which may be associated with severe clinical features and more extrapulmonary complications. This review focuses on the characteristics of *M. pneumoniae* infections in children, and exemplifies that simple clinical decision rules may help identifying children at high risk for CAP due to *M. pneumoniae*. This may aid physicians in prescribing appropriate first-line antibiotics, since current diagnostic tests for *M. pneumoniae* infection are not reliably predictive.

## Introduction

The clinical entity of “atypical” pneumonia was recognized in the 1930s many years before the etiological agent was established (McCoy, 1946). The term separated this entity of pneumonia from classical pneumococcal pneumonia due to its lack of response to available antibiotics and the distinct clinical presentation without typical lobar pneumonia and a less severe disease course. That is why the term “walking pneumonia” has been introduced to denote this mild form of pneumonia.

**FIGURE 1:**
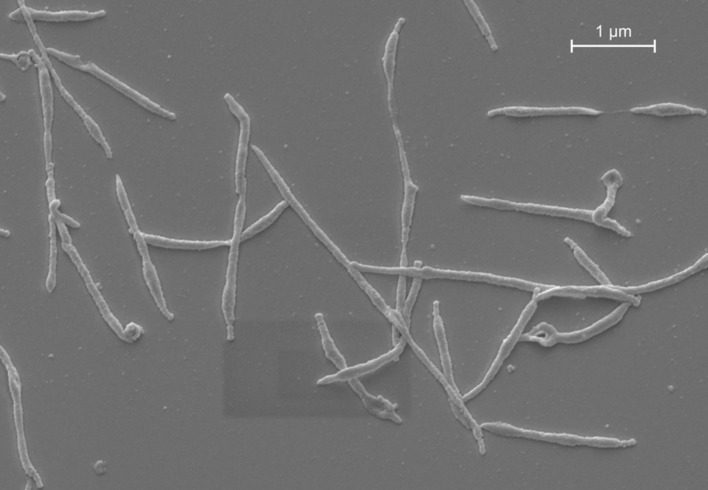
***M. pneumoniae* morphology *in vitro*.** Scanning electron micrograph of *M. pneumoniae* strain Mac (subtype 2).

It was in a patient with “atypical” pneumonia in 1944, where *Mycoplasma pneumoniae* was first isolated from sputum in tissue culture by Eaton et al. (1944). At that time, it was believed to be a virus because it was resistant to penicillin and sulfonamides and passed through bacteria-retaining filters. Experiments with Marine recruits and adult prisoners demonstrated that the so-called Eaton agent caused lower respiratory tract infections in humans (Chanock et al., 1961a,b). In 1963, it was first cultured on cell-free medium and classified as *M. pneumoniae* (Chanock et al., 1962; Chanock, 1963). Today we know that mycoplasmas are prokaryotes that lack a cell wall and represent the smallest self-replicating organisms (**Figure 1**). With a size of 816,394 base pairs, the genome of *M. pneumoniae* is at least five times smaller than that of *Escherichia coli* (Himmelreich et al., 1996). The absence of a cell wall and the specialized attachment organelle facilitate close contact with the host respiratory epithelium, which supplies the bacterium with the necessary nutrients for its growth and proliferation.

*Mycoplasma pneumoniae* causes both upper and lower respiratory tract infections, with community-acquired pneumonia (CAP) as the major burden of disease. Although *M. pneumoniae* infections are generally mild and self-limiting, patients of every age can develop severe and fulminant disease (Kannan et al., 2012). *M. pneumoniae* can also cause extrapulmonary manifestations that affect almost every organ (Narita, 2010).

In children, *M. pneumoniae* infections were first reported in 1960 when 16% of 110 children with lower respiratory tract disease were tested positive by a fourfold rise in antibody titers against the Eaton agent (Chanock et al., 1960). To date, it is known that the incidence of *M. pneumoniae* infections is generally higher in children than in adults (Foy et al., 1979). This review focuses on the characteristics of *M. pneumoniae* infections in children, and discusses simple clinical decision rules that may further aid clinicians in identifying patients at high risk for *M. pneumoniae* CAP.

## Epidemiology

*Mycoplasma pneumoniae* is transmitted by respiratory droplets through close contact. The incubation period can be long from 1 up to 3 weeks. Outbreaks have been reported within families, schools, universities, institutions, camps, and military bases. Family members of index patients with acute respiratory infection and detection of *M. pneumoniae* in the upper respiratory tract were found positive in 15% by polymerase chain reaction (PCR) (Dorigo-Zetsma et al., 2001). Thereof, 75% were <16 years of age and 44% did not develop any respiratory symptoms. At universities, the largest outbreak within 35 years in the U.S. was observed during September 1–December 4, 2012, where a total of 83 CAP cases were identified among students, and 12 out of 19 tested cases (63%) were positive for *M. pneumoniae* by quantitative real-time PCR (Centers for Disease Control and Prevention [CDC], 2013).

Outbreaks appear mainly during *M. pneumoniae* epidemics that occur in 3–7 years cycles, in addition to a background endemic pattern (Jacobs, 2012). The most recent epidemic in Europe occurred in 2010–2012 with a peak incidence in Finland of 145/100,000 cases in 2011 (Polkowska et al., 2012; Jacobs et al., 2015). The cyclic occurrence of epidemics may be facilitated by a decreasing herd immunity and different *M. pneumoniae* genotypes circulating in the human population (Jacobs, 2012). The two major circulating genotypes, or subtypes, of *M. pneumoniae* are indicated as subtype 1 and 2. Differences between these subtypes in the amino acid sequence of the major adhesion protein P1 are believed to play a role in the epidemiology of infections with *M. pneumoniae* (Su et al., 1990; Vink et al., 2012). The differences between the 169-kDa P1 proteins of subtype 1 and 2 isolates were found to be concentrated in two specific amino acid stretches within the protein. These regions are encoded by two DNA elements within the P1 gene, i.e., repetitive elements RepMP2/3 and RepMP4. The RepMP2/3 and RepMP4 are not unique to the P1 gene, but are also found at other sites within the bacterial genome (Spuesens et al., 2009). Homologous recombination events between these repetitive elements, which are similar to each other, but not identical, may form the basis of antigenic variation of the P1 protein of *M. pneumoniae* (Vink et al., 2012). While such recombination events may induce antigenic variation within subtype 1 or subtype 2 strains, *M. pneumoniae* strains cannot switch from one subtype to the other, as the entire set of RepMP elements found in one subtype differs significantly from those found in the other subtype. Moreover, changes in the proportion of the two subtypes of *M. pneumoniae* were not observed between 2003 and 2012 in Europe (Jacobs et al., 2015).

## Infection

### Respiratory Disease

Although CAP is the major burden of disease, milder clinical presentations of *M. pneumoniae* respiratory infections may be much more common than CAP. These include acute bronchitis and upper respiratory tract infections (Esposito et al., 2000, 2002). *M. pneumoniae* could be detected by PCR and/or serology in 24% of non-streptococcal pharyngitis cases (Esposito et al., 2002).

It is estimated that 3–10% of children with *M. pneumoniae* respiratory infection develop CAP and that <5% of CAP cases are severe enough to require hospitalization (Waites and Talkington, 2004). Between 1963 and 1975, *M. pneumoniae* was detected by culture of respiratory specimens and/or a fourfold titer rise in complement fixation test (CFT) in 15–20% of radiologically confirmed CAP cases in Seattle, U.S. (Foy et al., 1979). In subsequent etiological studies, *M. pneumoniae* accounted for 4–39% of the isolates identified by PCR and/or serology in children with CAP admitted to the hospital (Juven et al., 2000; Principi et al., 2001; Baer et al., 2003; Michelow et al., 2004). *M. pneumoniae* was first reported as the most common bacterial cause of CAP in children requiring hospitalization in a U.S. multicenter study from 2011 to 2012 in Nashville and Salt Lake City (Jain et al., 2015). In this study, *M. pneumoniae* could be detected by PCR in 178 (8%) out of 2179 cases with CAP, whereas *Streptococcus pneumoniae* was found in 79 cases (4%).

**FIGURE 2:**
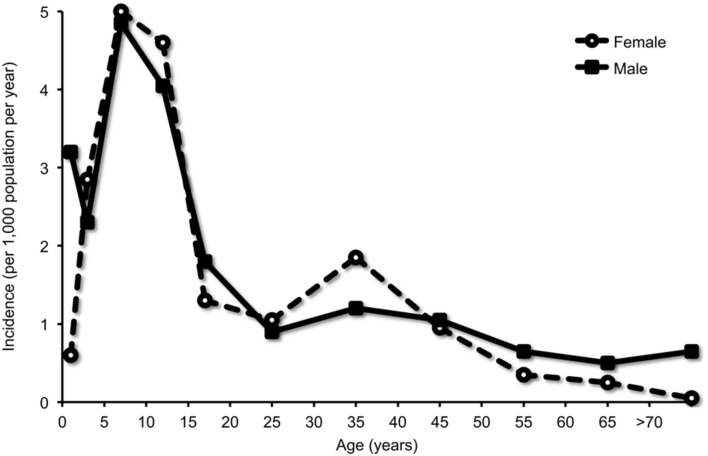
**Detection of *M. pneumoniae* in community-acquired pneumonia (CAP) according to age group.** Infection was diagnosed by culture of respiratory specimens and/or a fourfold titer rise in complement fixation test (CFT). Adapted with permission from Foy et al. (1979).

Manifest upper and/or lower respiratory tract infections with *M. pneumoniae* occur at all ages (Foy et al., 1979). Recent observations have indicated that *M. pneumoniae* has also a relatively high prevalence in the respiratory tract of children <5 years (Principi et al., 2001; Gadsby et al., 2012). *M. pneumoniae* CAP, however, was reported to be most frequent among school-aged children from 5 to 15 years of age, with a decline after adolescence and tapering off in adulthood (**Figure 2**) (Foy et al., 1979). This notion was corroborated in the recent CAP study in the U.S., where *M. pneumoniae* was detected significantly more frequent in children ≥5 years of age than in younger children (19% vs. 3%) (Jain et al., 2015).

In addition to the presentation at school-age, children with CAP due to *M. pneumoniae* have been found to present with a significantly longer duration of fever compared with other children with CAP (Fischer et al., 2002). Other symptoms that may be associated with *M. pneumoniae* CAP are the absence of wheeze and the presence of chest pain (Wang et al., 2012). However, there is still a paucity of high quality data regarding clinical signs and symptoms associated with *M. pneumoniae* infections. A recent Cochrane review therefore concluded that the absence or presence of individual clinical symptoms or signs cannot be used to help clinicians accurately diagnose *M. pneumoniae* in children and adolescents with CAP (Wang et al., 2012).

Pathogenic effects in the respiratory tract may be caused by *M. pneumoniae* either directly (by active infection), indirectly (by infection-induced immune mechanisms), or both (Narita, 2010). *M. pneumoniae* causes direct injury through the generation of activated oxygen. A potential candidate protein of *M. pneumoniae* that may be involved in causing direct damage to the respiratory tract is a pertussis toxin-like protein termed Community-Acquired Respiratory Distress Syndrome (CARDS) toxin (Kannan and Baseman, 2006; Becker et al., 2015). A recombinant version of the CARDS toxin has been shown to bind with high affinity to surfactant protein A and to exhibit mono-ADP ribosyltransferase and vacuolating activities, which causes disruption of the respiratory epithelium in animal models (Kannan and Baseman, 2006).

In addition to the direct damage resulting from infection by *M. pneumoniae*, the immunological response following infection generates inflammatory reactions that may cause pulmonary and extrapulmonary symptoms. More severe symptoms of CAP have been observed in older children and adolescents (Waites and Talkington, 2004). This suggests that the age-dependent magnitude and nature of inflammatory responses in childhood may be a major factor contributing to the development of *M. pneumoniae*-associated disease, similar to what is observed, e.g., in infectious mononucleosis or rheumatic fever. In fact, the severity of *M. pneumoniae* CAP in children was closely associated with increased concentrations of interleukin (IL)-8 and IL-18 in acute phase serum and pleural fluid samples (Narita and Tanaka, 2007). In addition, it has been demonstrated that cell-mediated immunity contributes to the pathogenesis of *M. pneumoniae* CAP, as it was shown that the severity of CAP correlated positively with the size of a cutaneous induration following intradermal injection of *M. pneumoniae* antigens (Mizutani et al., 1971). This study described 20 patients with CAP, of which 19 were children 4–15 years of age, diagnosed by a significant rise in antibody titers against *M. pneumoniae* with CFT. The strongest skin reactions were seen in patients with severe CAP.

### Asthma

*Mycoplasma pneumoniae* and other “atypical bacteria” have long been implicated in the pathogenesis of asthma (Atkinson, 2013). There are many studies that have addressed this issue in the recent past. In an observational study on children and adults with asthma, *M. pneumoniae* infection was diagnosed in 9% of children with asthma (24/256) and was found more frequent in patients with chronic asthma (14%) than in those with asthma exacerbations (7%; *p* = 0.10) (Bebear et al., 2014). The diagnosis of *M. pneumoniae* infection in this study was performed by PCR and/or serology. Another recent study diagnosed *M. pneumoniae* in children with acute asthma (64%, 34/53) and refractory asthma (65%, 17/26), as well as in healthy controls (56%, 36/64), but did not find significant differences between these three groups (Wood et al., 2013). The high detection rates reported in this study were obtained using novel diagnostic methods [CARDS toxin enzyme immunoassay (EIA) and CARDS gene-specific PCR] (Wood et al., 2013). In a recent Taiwanese study (Yeh et al., 2015), 1591 children and adults with *M. pneumoniae* infection, diagnosed by positive immunoglobulin (Ig) M or fourfold IgG titer increase, but without prior asthma history were included from 2000 to 2008 and followed until the diagnosis of asthma or the end of 2011. Compared to matched 6364 patients without *M. pneumoniae* infection, the cumulative incidence of asthma was significantly higher in the *M. pneumoniae* cohort than in the control cohort (*p* < 0.0001). Patients with *M. pneumoniae* infection were at higher risk of having early-onset asthma (age at asthma diagnosis <12 years) and late-onset asthma (age at asthma diagnosis ≥12 years). These most recent findings suggested that *M. pneumoniae* can induce airway inflammation and contribute to incident asthma. Interestingly, exposure to recombinant CARDS toxin resulted in an allergic-type inflammatory response and airway hyperreactivity in mice and baboons (Hardy et al., 2009; Medina et al., 2012). It will be interesting to investigate whether CARDS toxins induce a similar allergic response during *M. pneumoniae* infections.

### Extrapulmonary Manifestations

Apart from the respiratory tract infection, *M. pneumoniae* can cause extrapulmonary manifestations in almost every organ, including the skin and the hematologic, cardiovascular, musculoskeletal, and nervous systems (Narita, 2010). These manifestations may be caused either by direct local effects of *M. pneumoniae*, after dissemination of the bacteria throughout the body, or indirect effects, such as autoimmune reactions. The most frequent manifestations are diseases of the dermatologic and nervous system.

**FIGURE 3:**
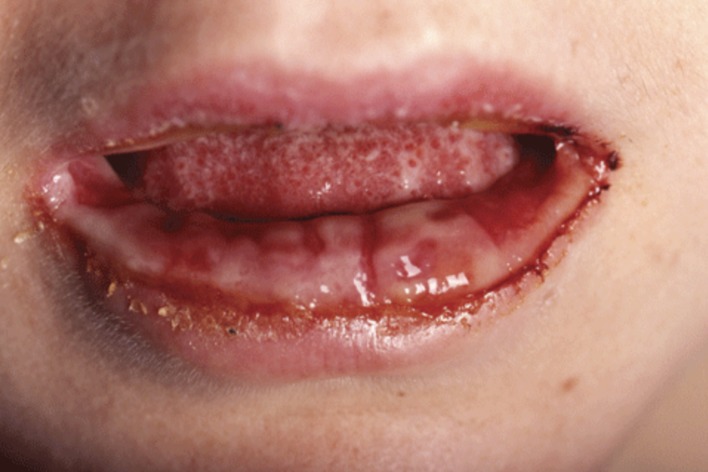
***M. pneumoniae*-associated mucositis (MPAM).** Erosive oral lesions limited to the mucosa in this form of MPAM in a 24-year-old woman. Reprinted with permission from Meyer Sauteur et al. (2012).

Skin manifestations occur in up to 25% of all *M. pneumoniae* infections, including mostly non-specific exanthems, erythema nodosum, urticaria, Stevens–Johnson syndrome, and a rare but distinct disorder with prominent mucous membrane involvement denominated as *M. pneumoniae*-associated mucositis (MPAM) (Schalock and Dinulos, 2009; Meyer Sauteur et al., 2012). This condition was first described by Fuchs (1876), and therefore also referred to as Fuchs syndrome (Meyer Sauteur et al., 2011). A recent review identified 32 patients with MPAM at a median age of 13.5 years at presentation (range 3–38 years, 23 children or young adolescents ≤18 years) (Meyer Sauteur et al., 2012). All patients presented with prodromal respiratory symptoms with a median duration of 7 days, and pneumonia was found in chest radiography in 79%. Oral lesions were present in all cases (**Figure 3**), ocular lesions in 97%, and urogenital lesions in 78%. There were no skin lesions in 69%. Although 12% of the patients were admitted to the intensive care unit, no one suffered from long-term sequelae.

**FIGURE 4:**
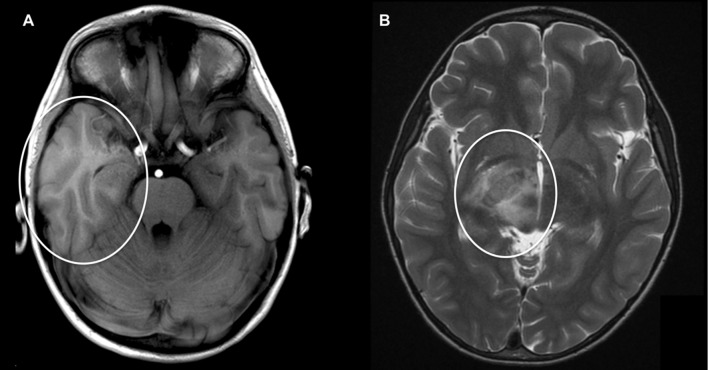
***M. pneumoniae*-associated encephalitis.** Axial cranial magnetic resonance imaging (MRI) in two children with encephalitis during *M. pneumoniae* infection: **(A)** 5-year-old boy with hyperintensity and generalized edema of the right temporal lobe [T1 weight MRI; patient 1 published in Meyer Sauteur et al. (2016)]. **(B)** 9-year-old boy with generalized edema of crus posterior of capsula interna [T2 weight MRI; reprinted with permission from Meyer Sauteur et al. (2014c)].

Encephalitis and Guillain–Barré syndrome (GBS) constitute the most common and severe neurologic manifestations, where *M. pneumoniae* infection is established in up to 10 and 15% of patients, respectively (Bitnun et al., 2001; Sinha et al., 2007). In *M. pneumoniae*-associated encephalitis, both a direct infection of the central nervous system (CNS) and an immune-mediated process have been implied to be involved (Narita, 2009). Because the detection rate of *M. pneumoniae* by PCR in cerebrospinal fluid (CSF) of *M. pneumoniae* encephalitis patients is relatively low (0–14%) (Bitnun et al., 2001; Daxboeck et al., 2004; Christie et al., 2007a; Domenech et al., 2009), a significant proportion of the cases is believed to be immune-mediated. This is supported by the finding that various cases with *M. pneumoniae* encephalitis in which bacterial DNA could not be detected in CSF had a more prolonged duration of respiratory symptoms before the onset of encephalitis (>5–7 days) (Bitnun et al., 2001; Narita and Yamada, 2001; Daxboeck et al., 2004). These cases indicate that *M. pneumoniae* encephalitis represents a postinfectious disorder, which manifests after clearance of the bacteria from the CNS or respiratory tract by the immune system (Meyer Sauteur et al., 2014b). A recent study presented 365 children with *M. pneumoniae* detected in the respiratory tract or CSF by PCR, 22 (6%) of whom had encephalitis (1996–2013, Toronto, ON, Canada) (Al-Zaidy et al., 2015). Interestingly, patients in which *M. pneumoniae* was detectable in the respiratory tract but not in CSF showed pulmonary infiltrates on chest radiograph more frequently than patients with positive PCR in CSF (77% vs. 33%). This suggests that pneumonia may be an indicator for a remote inflammatory process in *M. pneumoniae* encephalitis patients, which was also shown in 83% (5/6) of children observed during a national surveillance, all with negative PCR in CSF (2010–2015, Switzerland) (Meyer Sauteur et al., 2016) (**Figure 4**).

*Mycoplasma pneumoniae* expresses adhesion proteins and glycolipids that share structural homology with a variety of host cells (molecular mimicry) and may induce cross-reactive antibodies (Meyer Sauteur et al., 2014b). In children with *M. pneumoniae* encephalitis, intrathecal antibodies directed against galactocerebroside (GalC) were found (Christie et al., 2007b; Meyer Sauteur et al., 2015a). Of note, all these patients had a negative PCR in CSF. GalC is a major glycolipid antigen in the myelin sheath of both the peripheral and CNS neurons (Menge et al., 2005). In fact, antibodies against *M. pneumoniae* infection have been found to cross-react with GalC in GBS patients (Kusunoki et al., 2001; Ang et al., 2002). Moreover, anti-GalC antibodies caused demyelinating neuropathy in rabbits (Saida et al., 1979) and have been associated with demyelination in GBS (Ang et al., 2002), but also in encephalitis (Christie et al., 2007b) and encephalomyelitis (Samukawa et al., 2012). The detection of intrathecal antibodies against *M. pneumoniae* and GalC may also be regarded as a promising new diagnostic tool for *M. pneumoniae*-associated CNS disease (Meyer Sauteur et al., 2014b, 2015b).

## Carriage

Like many other respiratory pathogens, *M. pneumoniae* can be carried asymptomatically in the respiratory tract (Foy, 1993). Recent studies have demonstrated that asymptomatic carriage of *M. pneumoniae* is highly prevalent. Detection rates of *M. pneumoniae* DNA in the respiratory tract of healthy children without respiratory symptoms were 21% in a Dutch study (2008–2011, Rotterdam, The Netherlands) (Spuesens et al., 2013) and 56% in a U.S. study (2009–2011, San Antonio, TX, U.S.) (Wood et al., 2013). Longitudinal sampling of *M. pneumoniae*–positive asymptomatic children demonstrated that *M. pneumoniae* can be present in the upper respiratory tract without causing disease, for up to 4 months (Spuesens et al., 2013). The prevalence of *M. pneumoniae* in the upper respiratory tract of asymptomatic children varied considerably between years and seasons. For example, asymptomatic carriage rates of 3% and 58% were reported in the spring of 2009 and the summer of 2010, respectively (Spuesens et al., 2013). These data suggest that carriage follows an epidemic pattern. It is tempting to speculate that this fluctuation in prevalence is related to the cyclic epidemics of *M. pneumoniae* infections. Apart from *M. pneumoniae*, children were found to simultaneously carry many pathogens in their nose and throat (Spuesens et al., 2013). These pathogens include the bacteria *S. pneumoniae*, *Staphylococcus aureus*, *Moraxella catarrhalis*, and *Haemophilus influenzae*, and the viruses influenza A/B, human metapneumovirus, respiratory syncytial virus, parainfluenzavirus, rhinovirus, coronavirus, bocavirus, and adenovirus. The simultaneous presence of two or more of these pathogens was detected in 56% of asymptomatic children (Spuesens et al., 2013).

In children with *M. pneumoniae* CAP, co-existence of *M. pneumoniae* with other pathogens has also been described (Juven et al., 2000; Michelow et al., 2004), and was recently reported in 28% of the patients (Jain et al., 2015). The impact of co-infections in *M. pneumoniae* CAP on disease severity is not yet determined.

## Diagnosis

### Diagnostic Tests

Because the mere presence of *M. pneumoniae* in the upper respiratory tract is neither indicative nor predictive for respiratory disease, the routine diagnostic procedures to detect acute respiratory infections with *M. pneumoniae* need to be reconsidered. An overview of diagnostic tests with their advantages and drawbacks is shown in **Table 1**.

**Table 1:** Overview of diagnostic tests for *M. pneumoniae*.

Method	Test	Target/antigen	Antibodies	Specimen(s)	Performance	Value	Comments
Direct identification of *M. pneumoniae*	Polymerase chain reaction (PCR)	Different target genes (e.g., P1 gene, 16S rDNA, 16S rRNA, RepMP elements etc.)	–	Respiratory specimen Cerebrospinal fluid (CSF) Other bodily fluids or tissues	High sensitivity, high specificity	RD	- Validation and standardization required for routine diagnostic (Loens et al., 2010); - Epidemiological differentiation of clinical strains on the basis of differences in the P1 gene by PCR (Spuesens et al., 2009) or in the number of repetitive sequences at a given genomic locus by multiple-locus variable-number tandem repeat analysis (MLVA) (Chalker et al., 2015).
	Culture	–	–	Respiratory specimen	Low sensitivity, high specificity	AD	- Special enriched broth or agar media; - Isolation takes up to 21 days.
Non-specific serological tests for *M. pneumoniae*	Cold agglutinin test (“bedside test”)	Erythrocytes (I antigen)	Cold agglutinins (IgM)	Serum	Low sensitivity, low specificity	–^1^	- Cold agglutinins target the I antigen of erythrocytes; - Positive in only about 50% and in the first week of symptoms; - Less well studied in children; - Cross-reactivity with other pathogens and non-infectious diseases.
Specific serological tests for *M. pneumoniae*	Complement fixation test (CFT)	Crude antigen extract with glycolipids and/or proteins	Igs (no discrimination between isotypes)	Serum	Sensitivity and specificity comparable to EIA	–^1^	- Positive criteria: fourfold titer increase between acute and convalescent sera or single titer ≥1:32; - Cross-reactivity with other pathogens and non-infectious diseases.
	Particle agglutination assay (PA)		IgM and IgG simultaneously			–^1^	
	Enzyme immunoassay (EIA)	Proteins (e.g., adhesion protein P1) and/or glycolipids	IgM, IgG, IgA	Serum CSF^2^	Moderate-high sensitivity, Moderate-high specificity	RD	- The sensitivity depends on the time point of the first serum and on the availability of paired sera (for seroconversion and/or rise in titer); - “Gold standard”: fourfold titer increase as measured in paired sera.
	Immunoblotting				High sensitivity, high specificity	AD	- Confirmatory assay (Dumke et al., 2012).
	Immunofluorescent assay (IFA)				Less sensitive and less specific than EIA	AD	- Subjective interpretation.


*Adapted from Meyer Sauteur et al. (2014b). AD, advanced diagnostic test; CFT, complement fixation test; CSF, cerebrospinal fluid; EIA, enzyme immunoassay; IFA, immunofluorescent assay; Ig, immunoglobulin; PA, particle agglutination assay; PCR, polymerase chain reaction; RD, routine diagnostic test; RepMP, repeated M. pneumoniae DNA. ^1^Largely replaced by EIA; ^2^For the evaluation of an intrathecal antibody synthesis (Granerod et al., 2010), either by calculation of an antibody index (Reiber, 1994) or through parallel immunoblotting of simultaneously collected CSF and serum samples (Monteyne et al., 1997).*

Current guidelines (Bradley et al., 2011; Harris et al., 2011) recommend PCR and single-sample serological tests to diagnose *M. pneumoniae* infections. The sensitivity of serological tests depends on the time point of the first serum and on the availability of paired sera for seroconversion to IgG and/or rise in antibody titer. Specific serum IgM emerges within 1 week after initial infection and about 2 weeks before IgG (Meyer Sauteur et al., 2014b). Although specific serum IgA arises even earlier than IgM, it could be detected only in 2% of PCR-positive children with symptomatic respiratory tract infection (Spuesens et al., 2013). Cross-reactions with other pathogens and non-infectious disease has been described for CFT and particle agglutination assay, but also some EIAs lack the required sensitivity and specificity (Beersma et al., 2005). Further, it is intriguing that the detection of IgM, as well as IgG and IgA by EIA could not discriminate between the asymptomatic and symptomatic groups of children (Spuesens et al., 2013). The demonstrated positive serological results in asymptomatic *M. pneumoniae* PCR-positive children (*n* = 66; IgM in 17%, IgG in 24%, and IgA in 6%) may simply reflect one or more previous encounters with *M. pneumoniae* and are not necessarily related to the presence of *M. pneumoniae* in the respiratory tract. Thus, it is questionable whether or not a positive result in these tests actually indicates the etiological role of *M. pneumoniae* in all cases. In that sense, the positive predictive value of these tests may be overestimated, whereas the negative predictive value may be acceptable (Bradley et al., 2011).

The “gold standard” for diagnosis of *M. pneumoniae* infections is still considered to be a fourfold increase in antibody titer as measured in paired sera (Gardiner et al., 2015). However, the use of convalescent sera is not useful in clinical practice because it is too time-consuming and does not allow clinicians to initiate treatment protocols in a timely fashion. Clinicians therefore need to be aware of the implications and clinical significance of a positive PCR or serology test result.

### Clinical Assessment

While diagnostic tests may not be reliably predictive for a symptomatic *M. pneumoniae* infection, the clinical assessment of this entity is being revisited. The British Thoracic Society guidelines recommend that bacterial pneumonia should be considered in children when there is persistent or repetitive fever >38.5°C together with chest recession and a raised respiratory rate (Harris et al., 2011). A chest radiograph should not be considered a routine investigation in children thought to have CAP. In fact, although bilateral, diffuse infiltrates are common, none of the radiographic findings associated with *M. pneumoniae* CAP is specific (John et al., 2001).

**FIGURE 5:**
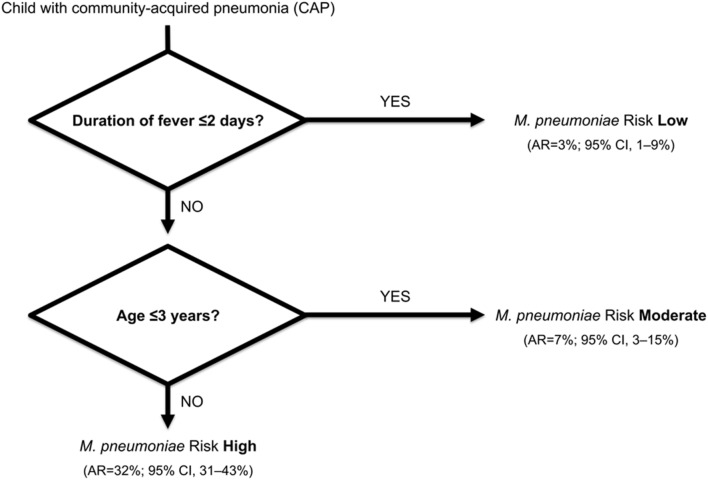
**A fast-and-frugal clinical decision tree for ruling out *M. pneumoniae* infection in children with community-acquired pneumonia (CAP).** Clinical features are considered sequentially, with a possible stop decision after each question. Abbreviations: AR, absolute risk; CI, confidence interval. Adapted from Fischer et al. (2002).

A fast-and-frugal clinical decision tree provided a rapid probability estimate of the cause of CAP in 253 children (1 months–16 years; 1997–1999, Zurich, Switzerland) (Fischer et al., 2002). *M. pneumoniae* infection was diagnosed in 13% (*n* = 32) of these children by PCR in respiratory specimens and serology (seroconversion and/or fourfold rise in antibody titer). Compared with other children with CAP, patients with *M. pneumoniae* were older and had a longer duration of fever (*p* < 0.001). Asking the simple question regarding the age of the child and the duration of fever allowed identification of the following group at high risk for CAP due to *M. pneumoniae*: children with CAP who have had fever >2 days and who were >3 years of age. The score model placed 75% of all patients with *M. pneumoniae* infection into the high-risk group (**Figure 5**). These simple rules may further aid physicians in prescribing appropriate first-line antibiotics.

## Treatment And Vaccines

### Antibiotics

In consequence of the diagnostic uncertainty for *M. pneumoniae* infections, the British Thoracic Society guidelines suggest empiric macrolide treatment at any age if there is no response to first-line β-lactam antibiotics or in the case of very severe disease (Harris et al., 2011). The lack of a cell wall makes *M. pneumoniae* resistant to cell wall synthesis inhibitors such as β-lactam antibiotics. The antibiotics with the best minimum inhibitory concentration values against *M. pneumoniae* include macrolides, tetracyclines, and fluoroquinolones (Waites and Talkington, 2004). Although the latter two have a good *in vitro* inhibitory effect against *M. pneumoniae*, tetracyclines may cause teeth discoloration (Waites and Talkington, 2004) and fluoroquinolones may affect the developing cartilage in young children (Adefurin et al., 2011). Thus, they are not recommended by current guidelines in young children; the age limit for tetracyclines is ≥8 years, while that of fluoroquinolones is adolescence with skeletal maturity (Bradley et al., 2011). The occurrence of arthropathy due to fluoroquinolones, however, is uncertain, and all musculoskeletal adverse effects reported in the literature had been reversible following withdrawal of treatment (Adefurin et al., 2011). The protein synthesis inhibitors of the macrolide class have a more favorable side effect profile and are therefore the first-line antibiotics for *M. pneumoniae* infections in children (Bradley et al., 2011).

Although antibiotics are effective against *M. pneumoniae in vitro* (Bebear et al., 2011), there is lack of evidence on their *in vivo* efficacy. Observational data indicated that children with CAP due to *M. pneumoniae* have a shorter duration of symptoms and fewer relapses when treated with an antimicrobial agent active against *M pneumoniae* (McCracken, 1986; Waites and Talkington, 2004). A recent Cochrane review evaluated seven studies on the effectiveness of antibiotic treatment for *M. pneumoniae* lower respiratory tract infections in children (Gardiner et al., 2015). However, the diagnostic criteria, the type and duration of treatment, inclusion criteria, and outcome measures differed significantly, making it difficult to draw any specific conclusions, although one trial suggested that macrolides may be efficacious in some cases (Esposito et al., 2005). It is clear that studies on the efficacy of antibiotics rely on a correct diagnosis of *M. pneumoniae* infections. Given the aforementioned shortcomings of current diagnostic tests, conclusions on the efficacy of antibiotic treatment will have to be re-examined.

### Antibiotic Resistance

**FIGURE 6:**
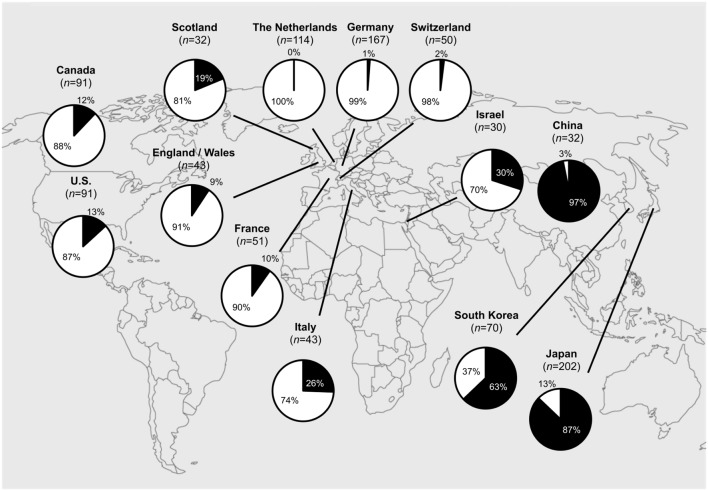
**Worldwide macrolide-resistant *M. pneumoniae* (MRMP) rates.** Actual MRMP rates are punctually depicted in pie charts (in black) over the world map. **Asia:** Japan (2011): 87% (176/202) (Okada et al., 2012), South Korea (2011): 63% (44/70) (Hong et al., 2013), China (2012): 97% (31/32) (Zhao et al., 2013), Israel (2010): 30% (9/30) (Averbuch et al., 2011); **North America:** U.S. (2012–2014): 13% (12/91) (Zheng et al., 2015), Canada (2010–2012): 12% (11/91) (Eshaghi et al., 2013); **Europe:** The Netherlands (1997–2008): 0% (0/114) (Spuesens et al., 2012), Germany (2003–2008): 1% (2/167) (Dumke et al., 2010), France (2005–2007): 10% (5/51) (Peuchant et al., 2009), Italy (2010): 26% (11/43) (Chironna et al., 2011), Scotland (2010–2011): 19% (6/32) (Ferguson et al., 2013), Switzerland (2011–2013): 2% (1/50) (Meyer Sauteur et al., 2014a), England and Wales (2014–2015): 9% (4/43) (Brown et al., 2015).

Since 2000, the extensive macrolide use led to an alarming worldwide increase in the prevalence of macrolide-resistant *M. pneumoniae* (MRMP) strains (Bebear et al., 2011). Resistance is based on specific point mutations in domain V of the *23S rRNA* (at positions 2063, 2064, and 2617), which reduce the affinity of macrolides to the large subunit (50S) of the bacterial ribosome (Bebear et al., 2011). MRMP has been observed during macrolide treatment as a result of antibiotic selective pressure (Cardinale et al., 2011; Chironna et al., 2011; Saegeman et al., 2012). To date, macrolide resistance has been detected on a worldwide scale. MRMP had developed in Asia (Hong et al., 2013), where MRMP rates have risen as high as 97% in China (Zhao et al., 2013). MRMP has now also been reported from North America and Europe (**Figure 6**).

The clinical relevance of macrolide resistance in hospitalized children with CAP may lie in prolonging the symptoms of the disease (Okada et al., 2012; Cardinale et al., 2013; Zhou et al., 2014). Zhou et al. (2014) found that an increase in MRMP may also have serious clinical consequences in children, leading to more severe radiological findings of CAP and even an increase in extrapulmonary manifestations. In this study, hospitalized children with CAP due to MRMP developed more often extrapulmonary disease than children with CAP caused by macrolide-sensitive strains (30% vs. 10%; *p* = 0.03) (Zhou et al., 2014). These manifestations included skin diseases and nervous system complications in 18% and 7%, respectively, of the MRMP-infected children. Serum inflammatory cytokine levels (INF-γ, IL-6, and IP-10) were higher in patients infected with MRMP than in patients infected with macrolide-sensitive strains (Matsuda et al., 2013). This suggests that the higher and more persistent inflammatory stimulation by MRMP may increase the possibility of severe lung lesions and extrapulmonary complications.

### Vaccines

While previous attempts to produce vaccines on the basis of inactivated bacteria resulted in limited efficacy against CAP and various adverse effects (Linchevski et al., 2009), the recent use of recombinant proteins as potential vaccines was found to be promising: The immunization of mice with a immunogenic recombinant protein encompassing the C-terminal part of the P1 protein (RP14) induced strong mucosal and systemic antibody responses against *M. pneumoniae*, and reduced lung inflammation in infected mice (Zhu et al., 2012). Another study showed that immunization of guinea pigs with a chimeric protein consisting of RP14 and the P30 adhesion protein of *M. pneumoniae* resulted in a robust antibody response that led to a reduction in bacterial loads in the respiratory tract (Hausner et al., 2013).

## Conclusion

The increasing prevalence of MRMP has become a significant issue, since MRMP can potentially cause more severe and even extrapulmonary disease. Because symptoms and radiologic features of *M. pneumoniae* CAP seem to be unspecific and current diagnostic procedures cannot discern between carriage and infection in a clinically relevant time frame, simple clinical rules may further aid physicians in prescribing appropriate first-line antibiotics. Thus, empiric macrolide treatment may be restricted to children at high risk for *M. pneumoniae* CAP, i.e., children with CAP who have fever >2 days and who are >3 years of age, or in the case of very severe disease. Future research should focus on novel aspects of *M. pneumoniae-*related pathogenesis resulting in more precise diagnostic tools and tailored treatment that prevents the emergence of antimicrobial resistance.

## Author Contributions

All authors listed, have made substantial, direct and intellectual contribution to the work, and approved it for publication.

## Conflict of Interest Statement

The authors declare that the research was conducted in the absence of any commercial or financial relationships that could be construed as a potential conflict of interest.
